# Spontaneous Bilateral Hyphema in a Patient With Idiopathic Thrombocytopenic Purpura

**DOI:** 10.7759/cureus.43505

**Published:** 2023-08-15

**Authors:** Spyros Atzamoglou, Ioannis Markopoulos, Evangelos Spanos, George Batsos, Vasileios Peponis

**Affiliations:** 1 Ophthalmology, Ophthalmiatreio Eye Hospital, Athens, GRC

**Keywords:** iop, hemorrhage, spontaneous, bilateral, itp, hyphema

## Abstract

The aim of this case report is to present an unusual case of idiopathic thrombocytopenic purpura (ITP) with bilateral spontaneous hyphema. It refers to an 82-year-old Caucasian woman who presented with acute unilateral vision loss. The patient’s medical history includes arterial hypertension, hypothyroidism, and uneventful bilateral cataract surgery. Bilateral anterior chamber hyphema was noted on gonioscopy, along with unilateral corneal edema. Hematology workup set the diagnosis of ITP. The cause of spontaneous bleeding in ITP patients is explained by the "second hit" hypothesis, suggesting that a secondary factor such as high blood pressure or minor trauma is necessary to cause rupture to a vessel’s wall, which is already affected by the low platelet counts. The authors propose that, in this patient, the "second hit" was likely due to basement membrane alterations caused by arterial hypertension. The rarity of bilateral spontaneous hyphema cases and possible etiologies are emphasized.

## Introduction

Acquired thrombocytopenia known as idiopathic thrombocytopenic purpura (ITP) is characterized by a platelet count below 100-109 K/μL and is caused by the immune destruction of platelets. ITP occurs in two to four per 100,000 adults and results in variable bleeding symptoms and thrombocytopenia and remains a diagnosis of exclusion [1].

Various mechanisms have been proposed to explain spontaneous bleeding in ITP [2]. The combination of low platelet count and damage to microvessel walls due to arterial hypertension or subclinical inflammation could be the cause of red blood cell (RBC) extravasation. The exact pathophysiology, however, remains to be determined [3,4].

We report an interesting case of spontaneous bilateral hyphema in an elderly Caucasian woman. This was the patient’s first and only symptom that led to the diagnosis of ITP. Although fatigue, petechiae, and epistaxis pose as routine findings in this syndrome, intraocular hemorrhage is rarely reported [5]. Common causes of intraocular hemorrhage include neovascularization of the anterior or posterior segment of the eye, uveitis, or trauma, although there are references to various types of intraocular hemorrhages due to hematologic disorders [6-10]. Interestingly, our patient presented only with bilateral spontaneous hyphema, which is exceedingly rare and, to the best of our knowledge, this is the first reported case in the setting of ITP.

## Case presentation

An 82-year-old Caucasian woman presented with unilateral acute vision loss in her right eye. The patient’s past medical history included arterial hypertension, hypothyroidism, and uneventful cataract surgery three years ago in both eyes. No history of ocular or other trauma was reported.

**Figure 1:**
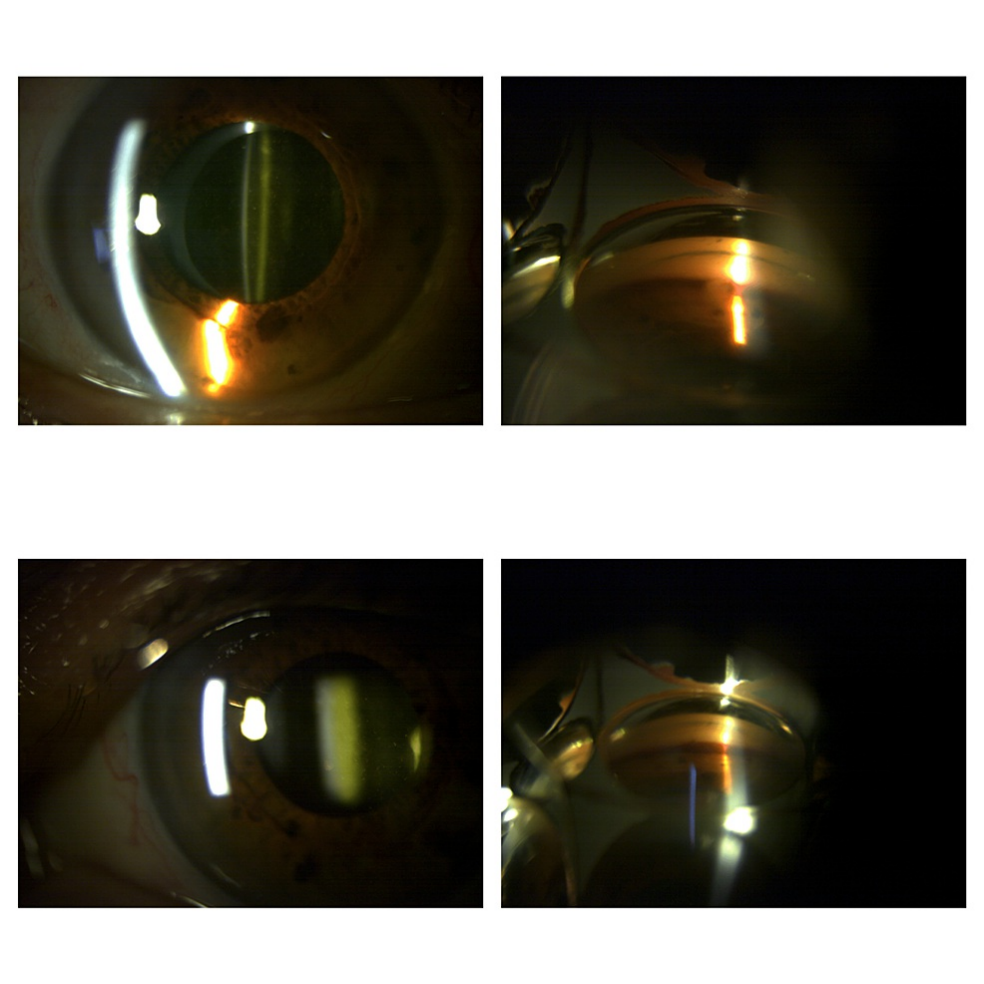
Bilateral hyphema. Note the grayish cells floating in each anterior chamber. A thin blood line is observed in the inferior quadrant on gonioscopy bilaterally.

On clinical examination, the best corrected visual acuity (BCVA) was 20/200 in the right eye (OD) and 20/25 in the left eye (OS). Bilateral pseudophakia was present in both eyes with IOLs located in the capsular bag. Intraocular pressure (IOP) was 58 mmHg and 42 mmHg in the right and left eye, respectively. Moderate conjunctival hyperemia, as well as mild corneal epithelial and stromal edema, were noted in the right eye. The left cornea was clear. Anterior chamber examination revealed bilateral reddish-greyish cells, with grade 3+ cells OD and grade 1+ cells OS. On gonioscopy, a deep anterior chamber angle (Shaffer Grade IV) was noted in all four quadrants (Figure 1), and hyphema was present in the lower quadrants bilaterally. Fundus examination was unremarkable in both eyes.

**Figure 2:**
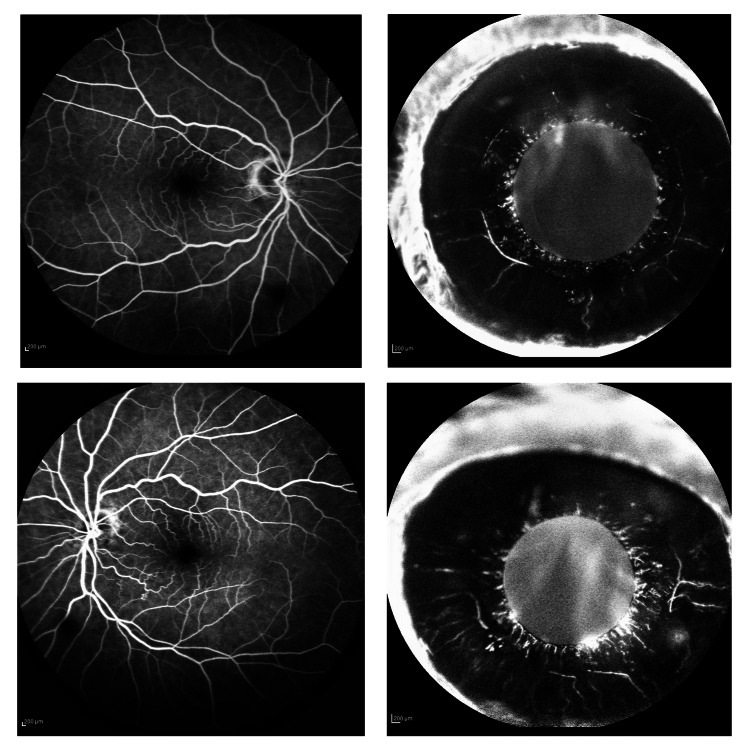
Fluorescein angiography. Fundus FA (OU) without significant findings. A dye leak can be seen originating from vessels in the pupillary border bilaterally, without any signs of neovascularization.

Topical dexamethasone, cyclopentolate, combined dorzolamide/timolol, apraclonidine, and per os acetazolamide 250 mg b.i.d were administered. During follow-up, corneal edema resolved, and IOP was reduced to 18 mmHg OD and 17 mmHg OS. BCVA improved to 20/25 bilaterally. Fluorescein angiography was performed and, although central and peripheral fundus findings were unremarkable, iris angiography revealed a dye leak in the anterior chamber originating from vessels at the pupillary border during the late phases of the examination (Figure 2). To our impression, the vessels appeared normal in configuration following a radial pattern, and no signs of neovascularization were detected [11]. Anterior and posterior segment ultrasound was performed without any significant findings.

A hematology workup with a full blood count revealed a very low platelet blood count (15 K/μL). Consultation from the Hematology Department was requested, and a peripheral blood smear confirmed the low platelet count. MRI scans, bone marrow biopsy, and full hematologic workup revealed no abnormalities, suggesting a diagnosis of ITP.

The patient was started on oral methylprednisolone, and when the platelet count normalized, spontaneous iris bleeding resolved bilaterally. Topical medication was discontinued. During follow-up, IOP remained within normal limits, with no signs of hyphema on gonioscopy. Three months after the initial presentation, the patient presented with a recurrence of the hyphema bilaterally, which was once again associated with low blood platelet count. Methylprednisolone dosage was adjusted accordingly, leading to the subsequent resolution of ocular findings.

## Discussion

ITP is an acquired thrombocytopenia, defined as a platelet count <100 K/μL, and caused by immune destruction of platelets in the absence of other causes or disorders that may be associated with thrombocytopenia. The diagnosis remains one of exclusion [1]. Platelet count above 30 K/μL has been linked to increased susceptibility to trauma-induced bleeding, while spontaneous bleeding has been associated with counts below 10-20 K/μL [2]. Various theories have been proposed to explain the so-called spontaneous bleeding in ITP through a seemingly uninjured vessel wall. Thrombocytopenia causes marked endothelium thinning and formation of wall fenestrations in capillaries and post-capillary venules [3]. However, patients with similar blood counts do not all bleed or present with varying bleeding severity. Additionally, bleeding in patients with thrombocytopenia is usually restricted to certain areas rather than being widespread [2].

As a result, a "second hit" hypothesis has been proposed in order to explain this phenomenon [2]. Fenestrations alone are not sufficient to cause rupture to the microvessels wall [2]. RBCs extravasate only through a damaged basement membrane. Thus, in addition to endothelium fenestrations, the necessary "second hit" could be a result of high blood pressure, inflammatory response, or minor trauma. Any of these factors could significantly contribute to the basement membrane rupture, allowing RBCs to extravasate [3,4]. Our hypothesis is that the "second hit" to our patient’s vascular structure is likely attributed to basement membrane alterations due to arterial hypertension.

The most common signs and symptoms of ITP include fatigue, petechiae, bleeding gums and epistaxis, hematuria, melena or rectal bleeding, profuse bleeding during surgery, and menorrhagia [5]. Ocular manifestations of ITP include sub-hyaloid, vitreous, and spontaneous supra-choroidal hemorrhage [12,13]. Hyphema in this case was the first and only sign of this hematologic abnormality. Immediate diagnosis and treatment in such cases are crucial as life-threatening bleeding could occur at any time.

Although cases of unilateral spontaneous hyphema have been described in previously published reports, there is a scarcity of bilateral acute spontaneous hyphema cases in the literature. Juvenile xanthogranuloma, warfarin use, iris microhemangiomas, and uveitis have been identified as possible etiologies [6,7,14-16]. Hematologic abnormalities, which have been associated with spontaneous bilateral hyphemas, are aplastic anemia and neonatal thrombocytopenia [8,9]. One case of unilateral hyphema due to chronic ITP has been reported by McDonald et al. [17].

## Conclusions

Concurrent bilateral spontaneous hyphema is an extremely rare finding, and clinicians should be aware of the possible causes. In ITP, addressing intraocular bleeding is as crucial as treating the underlying condition. In our case, this was the only manifestation of this life-threatening condition, and, to the best of our knowledge, this is the first case report of spontaneous acute bilateral hyphema due to ITP.
